# Population Dynamics and Biological Control of *Leucoptera malifoliella* in Apple Orchards in Hebei Province, China

**DOI:** 10.3390/insects17020171

**Published:** 2026-02-05

**Authors:** Jia-Qiang Zhao, Hong-Wei Zhang, Qi Gao, Sheng-Ping Zhang, Shi-Hang Zhao, Jian-Ming Li, Han Chang, Zhao-Hui Yang, Guo-Liang Xu

**Affiliations:** 1 Shijiazhuang Institute of Pomology, Hebei Academy of Agriculture and Forestry Sciences, Shijiazhuang 050061, China; jiaqiang_zhao@126.com (J.-Q.Z.); qigao977@haafs.org (Q.G.); zspingyouxiang@haafs.org (S.-P.Z.); zhaoshih3@haafs.org (S.-H.Z.); ljm1234554321@126.com (J.-M.L.); 17733508963@163.com (H.C.); 2 Chengde Agricultural Cash Crop Management Station, Chengde 067000, China; 13731419995@163.com; 3 Hebei Provincial Center of Agro-Product Safety and Quality, Shijiazhuang 050061, China

**Keywords:** *Leucoptera malifoliella*, population dynamics, sex identification, flight capacity, biological control

## Abstract

*Leucoptera malifoliella* has become a severe leaf-mining pest in Chinese apple orchards, especially under expanding organic and green cultivation practices, with effective management hindered by insufficient contemporary ecological data. To fill this gap, this 2023–2025 study conducted in Shijiazhuang, Hebei, combined field monitoring, morphological analysis, flight mill assays, and parasitoid release trials to clarify the moth’s phenology, develop rapid pupal sexing methods, quantify adult flight capacity, and assess *Trichogramma dendrolimi* biocontrol potential. The results showed five annual generations (overwintering as pupae), peak damage in July–August, and marked generational overlap. A reliable pupal sexing method was established via genital opening morphology. Adult flight peaked at 3 days post-emergence (max distance: 1.223 km), with no sexual dimorphism. Timely *T. dendrolimi* releases boosted parasitism rates, achieving 23.4–49.6% control efficacy during peak damage, with the parasitism rate positively correlated with efficacy. This study confirms the moth’s potential for generational increase under climate warming and medium-distance dispersal capacity, validating *Trichogramma’s* utility and laying a scientific foundation for precise, regionally coordinated ecological management.

## Simple Summary

Environmentally benign pest control is critical for apple orchards. The pear-leaf blister moth has emerged as a major pest, particularly in low-chemical-input orchards, yet modern ecological data on this pest remain insufficient for effective management. From 2023 to 2025, we examined this moth in Hebei apple orchards, monitoring population phenology, developing a rapid pupal sexing method, quantifying adult flight capacity, and evaluating *Trichogramma*-mediated egg parasitism. The results showed five annual generations, with peak damage in July–August; adults flew over 1.2 km, enabling inter-orchard dispersal. Timely *Trichogramma* releases enhanced egg parasitism and reduced leaf damage. This study indicates that the moth may proliferate under climate warming and that *Trichogramma* application is an effective, eco-friendly tactic, laying the groundwork for precise, ecological pest management and reduced chemical use.

## 1. Introduction

The apple tree (*Malus domestica* Borkh) is one of the world’s most economically important fruit crops [1,2]. Consumed fresh or processed year-round, it constitutes a vital economic pillar and a key component of food security in temperate regions [3,4]. In 2023, China’s apple production reached 49.6 million tons, accounting for 57.2% of global output and valued at approximately USD 23.4 billion. The widespread practice of fruitlet bagging has markedly suppressed internal-feeding pests like the codling moth (*Cydia pomonella* L.) and the oriental fruit moth (*Grapholita molesta* Busck) [5,6]. However, this practice has concurrently instigated a shift in pest complexes, elevating foliage-feeding insects, particularly leaf-mining Lepidoptera, to primary pest status [7]. The larval endophytic feeding habit within the leaf parenchyma makes these miners inherently cryptic, narrows the window for effective insecticide application, and facilitates the evolution of resistance. Their mining activity directly impairs photosynthesis by destroying palisade and spongy mesophyll, often leading to premature leaf abscission and creating entry points for secondary pathogens. The cumulative impact is a progressive decline in both yield and fruit quality [8,9,10].

**Figure 1:**
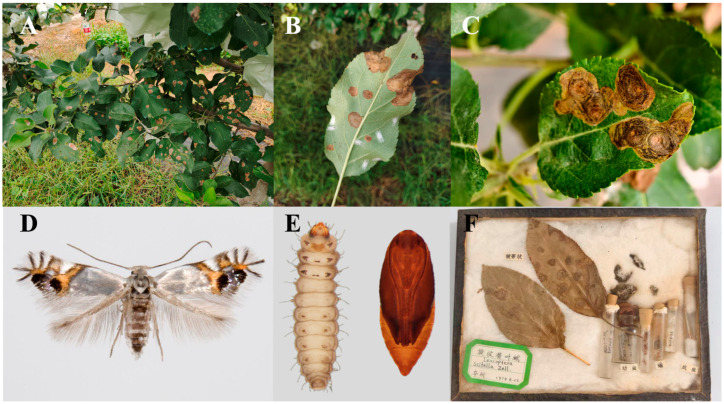
Photographs of *Leucoptera malifoliella*: (**A**–**C**) Damage to apple leaves; (**D**) adult; (**E**) larva and pupa; (**F**) a specimen prepared in 1979.

Among these pests, the pear-leaf blister moth, *Leucoptera malifoliella* O. Costa (Lepidoptera: Lyonetiidae), has become a key pest of Rosaceae fruit trees globally [11,12,13]. The larvae of this species construct distinctive spiral tunnels adorned with frass rings on apple, pear, hawthorn, and crabapple foliage while lining the interior of these galleries with a layer of prominent black frass (Figure 1A–E). Severe infestations can produce over ten mines per leaf, drastically reducing photosynthetic area, inducing premature defoliation, weakening tree vigor, and downgrading fruit quality [14,15].

Taxonomically, *L. malifoliella* was historically confused with *L. scitella* Zeller. Subsequent revisionary work established them as objective synonyms, with *L. malifoliella* holding nomenclatural priority [16]. All life stages are morphologically distinctive. Adults are small, with a body length of 2.3–2.7 mm and a wingspan of 6–8 mm. They appear silvery-white, with the apical two-thirds of the forewing being bright orange-yellow and marked with seven oblique brownish streaks, with the distal three to four radiating from the apex. Mature larvae measure 4–5 mm, are flattened and pale yellow-white, and possess a characteristic chestnut-brown, bilobed prothoracic shield. Pupae, approximately 3 mm long and elongate-oval (Figure 1D,E), are formed within a white, spindle-shaped cocoon. This cocoon is adorned with a unique H-shaped silk web, a structure hypothesized to offer protection against parasitoids and rainfall [17,18].

Native to Europe and Central Asia, the species’ current distribution extends from Western Europe (France and Germany) to China, Turkey, Iran, and Kazakhstan [19]. Foundational population ecology studies were primarily conducted in the late 20th century (Figure 1F), for instance, in Italy during the 1960s–1980s and in China during the 1960s–1990s [20,21,22]. The subsequent era of broad-spectrum insecticide application successfully suppressed its populations to secondary status. However, a confluence of factors—including increased winter survival rates, growing consumer demand for residue-free fruit, and the expansion of organic production—has triggered a notable resurgence [23,24,25,26]. For example, in 2022, organic orchards near Shijiazhuang, Hebei Province, reported up to 80% leaf damage. Furthermore, recent detections signal an ongoing geographical expansion: in 2024, the moth was first recorded in Tunisia (in Kasserine Province), colonizing six districts within months, indicating a southward spread across the Mediterranean [17]. In 2025, it was first documented in the Kashmir region of India, where it has since caused severe outbreaks characterized by high population densities, posing a significant new threat to local apple production [27].

Consequently, life-table data that are several decades old are now inadequate for designing precision pest management. Critical knowledge gaps persist, particularly concerning the current number of annual generations under modern climatic conditions, adult dispersal capacity, and interactions with local natural enemies. These gaps hinder the development of robust, ecology-based integrated pest management (IPM) strategies that minimize chemical reliance [15,28,29]. To address these deficiencies, we integrated systematic field monitoring with targeted laboratory assays in Shijiazhuang, Hebei Province. Our specific objectives were (i) to define the seasonal phenology and population dynamics of *L. malifoliella*; (ii) to develop a rapid, non-destructive method for sexing pupae; (iii) to quantify age- and sex-specific flight capacity using flight mills; and (iv) to evaluate the field efficacy of the indigenous egg parasitoid *Trichogramma dendrolimi* Matsumura for biological control. This study aims to fill these critical knowledge gaps, provide a foundation for a regional conservation-augmentation biological control system, and offer a forward-looking management framework applicable to areas grappling with this moth’s ongoing global expansion.

## 2. Materials and Methods

### 2.1. Study Site and Period

This study was conducted from April 2023 to October 2025 in a commercial apple orchard located in Chang’an District, Shijiazhuang City, Hebei Province, China (38.125° N, 114.526° E; 78 m above sea level). The orchard was planted with 8- to 15-year-old ‘Fuji’ apple trees (*M. domestica* ‘Fuji’) managed according to standardized horticultural practices. Crucially, no insecticides were applied throughout the study period to avoid interference with natural population dynamics and parasitoid activity. The region experiences a temperate continental monsoon climate characterized by an annual average temperature of 12.9 °C and annual precipitation of 536.8 mm, with approximately 70% of rainfall occurring between June and August. The frost-free period lasts approximately 200 days.

### 2.2. Field Population Dynamics Monitoring

A randomized block design was implemented across the experimental orchard. Six monitoring stations were established with inter-station distances of ≥100 m to ensure spatial independence. At each station, five apple trees of uniform growth vigor and age were permanently designated as fixed observation units.

Systematic surveys were conducted three times weekly from April through November. On each observation tree, four representative branches (30–50 cm in length) were selected at the cardinal compass points (east, south, west, north) within the 1.0–2.0 m height stratum—corresponding to the mid-to-low canopy zone where *L. malifoliella* infestation is most prevalent. Twenty leaves were randomly examined per branch (yielding 80 leaves per tree; 400 leaves per monitoring point per survey).

During each inspection, we recorded the presence and abundance of all *L. malifoliella* life stages (eggs, larvae, pupae, and adults) and quantified leaf mine incidence. To prevent pseudoreplication across surveys, leaves bearing old, desiccated mines (indicative of previous generations) were marked with distinct color codes using permanent markers upon first detection and excluded from subsequent counts; only fresh, active mines containing live larvae were recorded as new damage.

### 2.3. Rapid Identification of the Sex of Pupae

During the peak occurrence period of *L. malifoliella* (July–August), apple branches (with leaves) bearing pupal cocoons were carefully collected from trees outside the fixed observational plots and transported to the laboratory. Samples were maintained in rearing cages (40 × 30 × 30 cm) inside a climate-controlled chamber set at 25 ± 1 °C, 70 ± 5% relative humidity (RH), and a 14:10 h (L:D) photoperiod. Absorbent cotton soaked in a 5% honey-water solution was provided as a nutritional supplement for emerging adults. For sex identification, the outer silk layer of the cocoon was carefully removed using fine forceps and scissors to extract the intact pupa. Each pupa was placed ventral side up on a glass slide. The morphological features of the abdominal terminus, particularly the eighth and ninth segments, were systematically examined and recorded under a stereomicroscope (SMZ25, Nikon, Tokyo, Japan). Fifty pupae were randomly selected, individually numbered, and assessed. Following morphological sex determination, these pupae were returned to the rearing cages under the same controlled conditions until adult emergence. The accuracy of the pupal sexing method was subsequently validated by examining the external genitalia of the adults that emerged.

### 2.4. Flight Capacity Assay

Flight capacity was measured using a computer-linked flight mill system (FXMD-24-USB, Jiaduo, Hebi, China). The system recorded flight parameters—total flight distance, total flight duration, and average speed—in real-time via dedicated software. Newly emerged adults were collected and aged to 1, 2, 3, and 5 days post-emergence (dpe) under standard rearing conditions. For each age cohort, 30 adults (15 females and 15 males, determined upon emergence) were tested. Prior testing, adults were mildly anesthetized by brief exposure to 4 °C for 2–3 min. A fine copper wire (diameter 0.1 mm) was attached to the center of the pronotum using a minute droplet of non-toxic adhesive and then tethered to the balanced arm of the flight mill. All tests were conducted in a dark environmental chamber maintained at 25 ± 1 °C and 70 ± 5% RH to eliminate visual stimuli. Flight activity was recorded continuously for 12 h. Following the criterion established by Stewart and Gaylor (1991), an individual was considered to have performed a ‘valid flight’ only if its total flight duration exceeded 10 min [30]. Individuals that failed to meet this threshold, or that died, became detached, or experienced technical issues during the test, were excluded from subsequent data analysis.

### 2.5. Assessment of Trichogramma Parasitoid Efficacy

The biological control potential of the egg parasitoid *T. dendrolimi* was assessed using commercially available parasitoid cards (Keyun^®^, Jiyuan, China). Each card contained approximately 15,000 parasitized *Corcyra cephalonica* Stainton eggs with viable *T. dendrolimi* pupae nearing emergence. Cards were stored at 4 °C prior to use and acclimated at ambient room temperature for 2 h before field release. A completely randomized block design was implemented within the orchard. Three independent release plots (0.5 ha each) and three control plots (0.5 ha each) were established. Release and control plots were spatially paired, with a minimum buffer distance of 100 m between plot pairs to minimize the risk of parasitoid drift into control areas.

The initial release of *Trichogramma* was carried out on 6 June 2025, coinciding with the peak oviposition period of the first summer generation of *L. malifoliella*, at a density of 112,500 individuals per hectare. A second release at the same density was implemented five days later (11 June), resulting in a cumulative release rate of 225,000 individuals per hectare. During deployment, parasitoid cards were evenly distributed across the experimental plots, with one card affixed to the shaded side of each tree trunk.

Systematic surveys were also conducted three times weekly, following a standardized five-point sampling method in each experimental plot. During each sampling event, 100 leaves were examined in situ to calculate the leaf damage rate, defined as the percentage of leaves harboring one or more active mines. Meanwhile, 50 leaves with fresh *L. malifoliella* mines were collected from both the release and control plots respectively, then transported to the laboratory and reared in ventilated containers. After 7–10 days, the parasitism status of larvae inside the mines was recorded.

The parasitism rate was calculated as follows:

Parasitism rate (%) = [Number of larvae parasitized by *Trichogramma*/Total number of larvae in the sampled mines] × 100

The relative control efficacy (RCE) was calculated as follows:

RCE (%) = [1 − (Leaf damage rate in release plots/Leaf damage rate in control plots)] × 100

### 2.6. Data Analysis

All statistical analyses and data visualization were performed using R software (version 4.3.0). Prior to parametric analyses, data distributions were tested for normality using the Shapiro-Wilk test and for homogeneity of variances using Levene’s test. The results of group comparisons were visualized using violin plots, which combine box plot summaries with kernel density estimation to effectively illustrate data distribution, probability density, and multimodality. For comparisons among multiple groups (e.g., flight parameters across different ages), one-way analysis of variance (ANOVA) was applied. When ANOVA results were significant (*p* < 0.05), Duncan’s multiple range test was used for post-hoc pairwise comparisons. For flight capacity data, a two-way ANOVA was employed to analyze the main effects of adult age, sex, and their interaction. Where no significant interaction was found, main effects were interpreted independently. Independent samples *t*-tests were also used for direct male-female comparisons within age groups. Effect sizes were reported using η^2^ (eta-squared) for ANOVA and Cohen’s d for *t*-tests. The relationships between parasitism rate, damage rate in release plots, and relative control efficacy (RCE) were analyzed using Pearson correlation and simple linear regression. The significance level for all statistical tests was set at α = 0.05.

## 3. Results

### 3.1. Seasonal Occurrence Patterns of L. malifoliella

**Figure 2:**
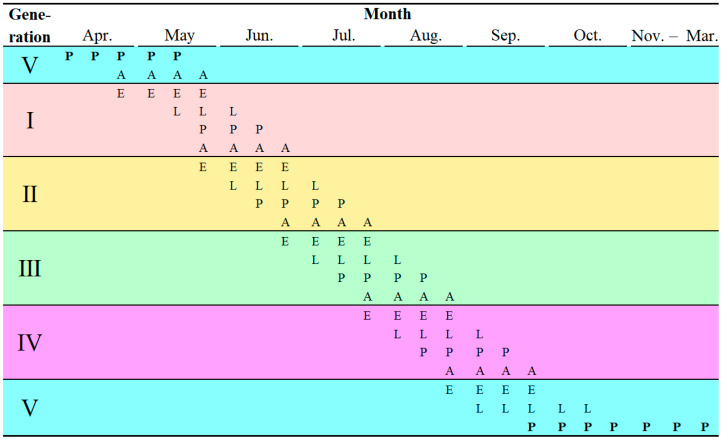
Schematic diagram of the life cycle of *Leucoptera malifoliella* in Shijiazhuang, China. A = adults; E = eggs; L = larvae; P = pupae; **Bold P = overwintering pupae**. Roman numerals I–V indicate the first through fifth (overwintering) generations.

Three consecutive years of systematic field monitoring (2023–2025) delineated the seasonal phenology of *L. malifoliella* in a commercial apple orchard in Chang’an District, Shijiazhuang. The pest completes five full generations per year in this area (Figure 2). Overwintering occurs in the pupal stage, with pupae located in bark crevices, under loose bark scales, and in tree branch forks.

Adults of the overwintering generation began emerging in late April. The first sign of larval activity—fresh leaf mines—was observed in early May, marking the onset of the first generation. First-generation adults emerged and initiated oviposition in early June. Notable generational overlap was apparent from the second generation onwards. A substantial emergence of second-generation adults occurred in early July. The peak emergence of third-generation adults followed in early August, which corresponded precisely with the period of maximum leaf damage observed in the orchard. This period of high pest activity spanned approximately two months. Fourth-generation adults began emerging in early September, with population densities declining thereafter as ambient temperatures dropped. The developmental duration of each generation was strongly temperature-dependent. The first (spring) generation had the longest life cycle (approximately 45 days), whereas the summer (second and third) and early autumn (fourth) generations exhibited significantly shorter cycles, ranging from 28 to 35 days. In late September, fifth-generation (overwintering) larvae ceased feeding, spun silken threads to descend from leaves, crawled to sheltered overwintering sites (bark crevices, etc.), spun their characteristic H-web cocoons, and pupated. The overwintering pupal stage lasted approximately seven months.

**Figure 3:**
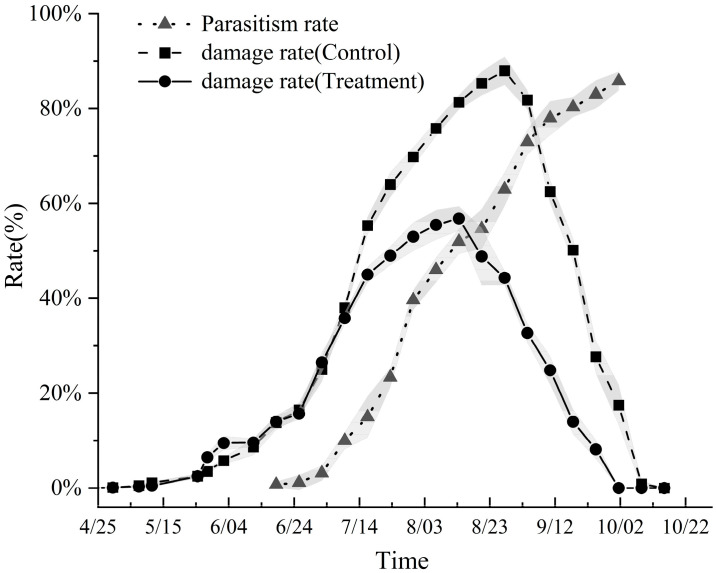
Seasonal progression of leaf damage rate (caused by *Leucoptera malifoliella*) and parasitism rate (by *Trichogramma dendrolimi*) in release and control plots during 2025. Shaded areas indicate the range (max–min) across three replicate plots, with solid lines representing mean values.

Seasonal population trends indicated exponential growth from June through August (Figure 3). In unmanaged control plots, peak infestation resulted in over 80% of leaves exhibiting mines. Field observations noted that adult eclosion occurred predominantly in the morning hours. Adults were largely quiescent during the day, resting on the undersides of leaves or on the leeward side of branches. Their flight was typified by short, spiraling bursts, primarily associated with oviposition and localized dispersal within the tree canopy.

### 3.2. Sexual Dimorphism in the Pupal Abdominal Terminus

**Figure 4:**
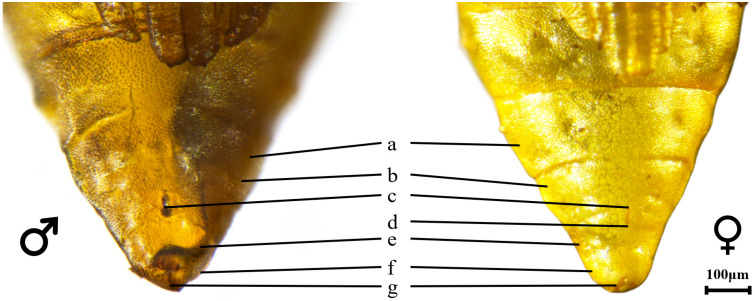
Morphological details of the abdominal terminus for sex differentiation in *L. malifoliella* pupae. (a) 7th abdominal segment, (b) 8th abdominal segment, (c) Gonopore, (d) Oviposition hole, (e) 9th abdominal segment, (f) 10th abdominal segment, (g) Anus. (**Left**) Male pupa; (**Right**) Female pupa.

Detailed morphological examination of the pupal abdominal terminus enabled the development of a reliable, non-invasive sexing technique for *L. malifoliella*. The primary diagnostic differences between male and female pupae are concentrated on the eighth and ninth abdominal segments (Figure 4). In female pupae, the gonopore (future ovipore) is situated closer to the anterior margin of the eighth segment and presents as a distinct longitudinal slit. In male pupae, the gonopore is located more centrally on the ninth abdominal segment and appears as a small, circular or slightly oval opening.

**Figure 5:**
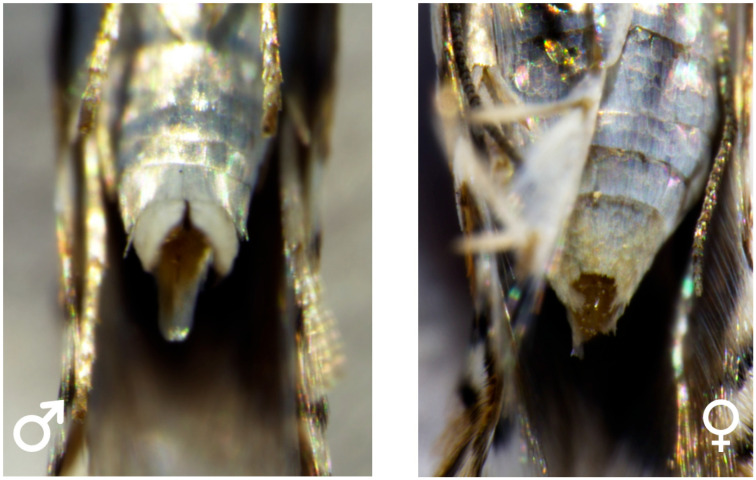
Ventral view of adult *Leucoptera malifoliella* highlighting external genitalia for sex confirmation. (**Left**) Male; (**Right**) Female.

The accuracy of the rapid sexing method relying on the morphological characteristics of the pupal abdominal terminus was verified via post-collection rearing and subsequent observation of the adults’ external genitalia after emergence. Sexual dimorphism in adults is primarily manifested in abdominal morphology (Figure 5): males have slender abdomens with distinctly prominent valvae at the posterior end, whereas females possess rounder abdomens than males. These adult sexing results confirm that the sex of *L. malifoliella* pupae can be accurately discriminated based on the morphological differences in their abdomens.

### 3.3. Flight Capacity Analysis

#### 3.3.1. Effect of Adult Age on Flight Capacity

**Figure 6:**
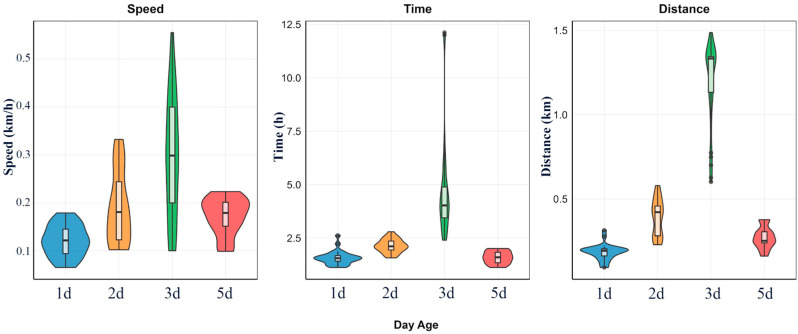
Comparison of flight parameters evaluated on a flight mill for *Leucoptera malifoliella* adults at different days post-emergence (dpe). Violin plots illustrate the data distribution, where the width (shape) indicates the probability density (frequency) of observations, and the vertical length represents the range of parameter values.

Systematic flight mill assays involving 101 valid adults (53 males, 48 females) demonstrated that adult age profoundly influenced the flight capacity of *L. malifoliella* (Figure 6). One-way ANOVA confirmed that age had a highly significant effect on all measured parameters: flight speed (*F* = 22.37, *df* = 3, *p* < 0.001), total flight duration (*F* = 37.94, *df* = 3, *p* < 0.001), and total flight distance (*F* = 247.82, *df* = 3, *p* < 0.001). All effect sizes (η^2^) exceeded 0.14, underscoring age as a major determinant of flight performance.

The relationship between flight parameters and age was unimodal, increasing to a distinct peak before declining. Both mean flight speed and total flight distance reached their maximum at 3 days post-emergence (dpe) (speed: 0.290 ± 0.138 km/h; distance: 1.223 ± 0.196 km). Values at 3 dpe were significantly greater than those at 1, 2, and 5 dpe (*p* < 0.05, Duncan’s test). The maximum mean flight distance at 3 dpe was 6.6 times greater than that of 1-dpe adults. Total flight duration showed a slightly different pattern, increasing progressively to a maximum at 3 dpe (4.43 ± 2.83 h) before a significant drop at 5 dpe. By 5 dpe, all flight parameters had regressed to levels statistically comparable to those observed at 1 and 2 dpe.

#### 3.3.2. Effect of Sex on Flight Capacity

**Figure 7:**
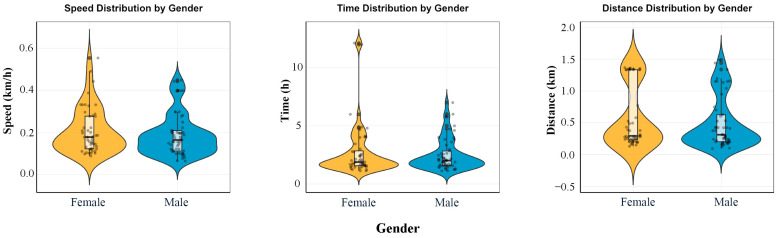
Comparison of flight parameters between male and female *Leucoptera malifoliella* adults. Violin plots depict the data distribution, with horizontal width representing observation density and vertical length indicating the value range. No significant differences were found (*p* > 0.05, *t*-test).

Contrary to our initial expectation, the sex of the moth did not significantly affect its flight capacity within the tested age range (Figure 7). Two-way ANOVA revealed no significant main effect of sex on flight speed, duration, or distance (*p* > 0.05 for all), and no significant interaction between sex and age was detected. Independent samples *t*-tests conducted within each age cohort further corroborated this finding, showing no statistically significant differences between males and females in flight speed (*t* = −1.12, *df* = 99, *p* = 0.267), total flight duration (*t* = −0.66, *df* = 99, *p* = 0.509), or total flight distance (*t* = −1.02, *df* = 99, *p* = 0.311) when data were pooled across ages.

Effect size analysis using Cohen’s d provided additional support; the absolute values of d for all flight parameter comparisons were below 0.3, falling within the range of a ‘small’ effect, indicating that any observed numerical differences were biologically negligible.

### 3.4. Efficacy Assessment of T. dendrolimi

Analysis of the full-season monitoring data revealed distinct temporal patterns in pest damage and parasitoid-mediated suppression (Figure 3). In control plots, the leaf damage rate increased rapidly from near-zero levels in late April, peaked sharply in late August (86.7 ± 4.3%), and then declined gradually, approaching zero by early October as larvae entered the overwintering generation.

In *Trichogramma* release plots, the damage rate initially tracked that of the control plots. However, following the first detection of *T. dendrolimi* activity on June 19 and a consistent increase in parasitism rate from late June onwards, the rise in damage rate was significantly attenuated. The peak damage rate in release plots (56.2 ± 4.2% in early August) was not only significantly lower (*p* < 0.05) than the concurrent peak in paired control plots (78.6 ± 4.6%) but also occurred slightly earlier. Most importantly, as the parasitism rate entered a sustained high plateau in late August (58.8 ± 8.8%) and continued climbing to its seasonal maximum in late September (84.4 ± 4.4%), the damage rate in release plots began a rapid and premature decline. It fell below 20% by mid-September, a time when damage rates in control plots remained elevated at 56.3 ± 8.7%.

The temporal trajectory of the Relative Control Efficacy (RCE) clearly delineated the period of effective biological control. Beginning in late June, as the released parasitoid population established itself, RCE values increased rapidly and remained at a high, stable level throughout July and August, averaging between 23.4% and 49.6% during this peak pest period. Notably, in mid-to-late September, while damage rates in control plots were still significant, the RCE in release plots continued to increase due to the precipitous drop in damage, reaching a peak of 60.4%. This confirms a sustained control advantage imparted by *T. dendrolimi*. The field trial demonstrated that augmentative releases exerted significant and durable suppression on *L. malifoliella* populations.

**Figure 8:**
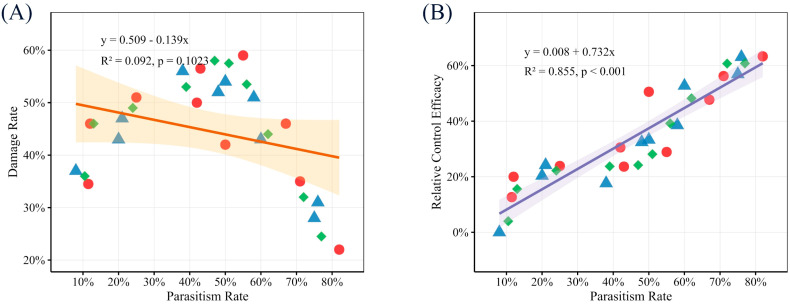
Linear regression relationships between *Trichogramma* parasitism rate and (**A**) *Leucoptera malifoliella* damage rate, and (**B**) relative control efficacy. Red circles, blue triangles, and green diamonds represent data from Replicates 1, 2, and 3, respectively. Solid lines denote the fitted linear regression curves (with shaded areas indicating 95% confidence intervals).

Correlation and regression analyses provided mechanistic insight. A highly significant positive linear correlation was found between the field parasitism rate and the relative control efficacy (RCE) (r = 0.925, *p* < 0.001) (Figure 8B). Linear regression indicated that for every 10% increase in parasitism rate, the RCE was predicted to increase by approximately 7.3% (R^2^ = 0.855). This quantitatively validates that suppression of the effective pest population via enhanced parasitism is the core mechanism driving control. Conversely, the relationship between parasitism rate and the absolute damage rate in release plots showed a negative but non-significant trend (r = −0.304, *p* = 0.102) (Figure 8A), suggesting that while parasitism directly reduces pest numbers, its translation into immediate visible damage reduction may be less direct or confounded by other factors.

## 4. Discussion

### 4.1. Population Dynamics in the Context of Climate Change

Our documentation of five annual generations for *L. malifoliella* in Northern China represents a significant departure from historical reports of one to four generations in temperate Europe [11]. This shift strongly indicates a climate-mediated acceleration in phenology. The extended adult activity window (April to October) coupled with pronounced generational overlap creates near-continuous pest pressure, rendering traditional calendar-based spray schedules increasingly ineffective. Our data underscore the critical importance of intervention prior to the exponential population growth phase in July–August, providing concrete evidence for optimizing the timing of control tactics.

The primary driver behind this increased generation number is likely the cumulative effect of elevated effective accumulated temperatures (EATs) [31]. The observed lengthening of the physiologically favorable activity period, combined with potentially greater overwintering survival under milder winters, provides the necessary temporal and physiological framework for completing an additional generation. This aligns with broader patterns of insects’ phenological responses to global environmental change. Supporting evidence comes from regional climate trends; for instance, Jung et al. noted that the warming rate in Korea (1.8 °C from 1912–2010) far exceeded the 20th-century global average, leading to projections that the annual generation number of the apple leafminer *Phyllonorycter ringoniella* Matsumura could increase significantly, potentially reaching 6.7 generations by the 2090s [32]. Such generational proliferation inevitably intensifies management pressure. Theoretical models for estimating generation number under warming scenarios, such as that by Yamamura and Kiritani (1998), provide a robust framework for interpreting our empirical findings [33].

Comparisons with recent studies conducted elsewhere highlight this species’ expansive potential. Hulujan et al. reported three complete generations in Romanian cherry orchards, while Soltani and Rahmouni documented its rapid establishment across six Tunisian districts within two years of initial detection [17,34]. These instances collectively demonstrate a robust adaptability to diverse climatic zones. This expansion pattern mirrors the response of many polyphagous pests to global warming. As demonstrated in a meta-analysis by Liu et al. on *Helicoverpa armigera* Hübner, key physiological and developmental rates are significantly optimized at temperatures above 20 °C, peaking between 32–35 °C [35]. This thermal plasticity explains how species once confined to specific temperate ranges can progressively breach historical geographical barriers.

### 4.2. Dispersal Ecology and Management Implications

Our study provides the first precise quantification of *L. malifoliella* adult flight capacity, identifying a distinct post-emergence maturation period peaking on day 3, with a maximum recorded distance of 1.223 km. This defines a “medium-distance dispersal” capability (1–2 km), which has profound implications for management. This range readily permits movement across individual orchard boundaries, drastically reducing the efficacy of isolated, single-orchard control measures. This stands in sharp contrast to the ultra-long-distance migratory capacity documented for species like *Lymantria xylina* Swinhoe [36], highlighting the interspecific variability in dispersal strategy. Notably, the moth’s typical flight range closely mirrors the common spacing between orchard patches in agricultural landscapes (0.5–2 km), thereby elevating the risk of rapid colonization and area-wide spread. Consequently, our findings strongly advocate for a paradigm shift from isolated “single-orchard control” towards areawide, landscape-coordinated pest management.

The lack of significant sexual dimorphism in flight capacity was unexpected but may be attributable to generally similar body size and resource allocation patterns between sexes in this species. Similar patterns have been noted in other Lepidoptera, such as the fall armyworm (*Spodoptera frugiperda* Smith), where flight distance and duration showed no sexual difference, though speed differences were sometimes observed [37]. This contrasts with species like the spongy moth (*L. dispar* L.), where females are stronger fliers [38,39], or others where males disperse more seeking mates. The several-day pre-peak flight period post-emergence is ecologically significant. It opens a critical “management window” for implementing control barriers (e.g., mating disruption, targeted insecticide applications, or biological releases) before the population achieves its full dispersal potential. While autonomous flight is moderate, the small size and low weight of adults make them highly susceptible to passive long-distance dispersal via human-assisted transport (e.g., on plant material) or wind currents [7,40], a risk factor compounded by its broad host range [17].

Therefore, effective management strategies must explicitly account for landscape connectivity. We propose the establishment of coordinated management zones with a recommended radius of 1–2 km. Within these zones, unified monitoring, synchronized action thresholds, and coordinated application of multiple control tactics (chemical, biological, cultural) should be implemented. This landscape-scale, cooperative approach, informed by a solid understanding of dispersal ecology, is essential for mitigating the spread and impact of *L. malifoliella*.

### 4.3. Challenges and Opportunities for Biological Control

Field trials confirmed that augmentative releases of *T. dendrolimi*, when precisely timed to coincide with the first summer egg peak (early June), can allow sustained suppression of *L. malifoliella*, achieving approximately 60% relative control efficacy (RCE) by late season. The strong correlation between parasitism rate and RCE (R^2^ = 0.855) underscores that successful control depends fundamentally on phenological synchrony—ensuring high natural enemy abundance precedes the pest’s most rapid population growth [41].

While reduced pesticide use in IPM systems promotes natural enemy conservation [42] and enhanced orchard biodiversity supports more resilient enemy networks [43], several constraints limit the reliability of biological control. Efficacy remains sensitive to abiotic variables such as temperature and humidity, and extreme weather events associated with climate change can disrupt critical synchrony between host and parasitoid. Additionally, competition among natural enemy species, or between strains, may sometimes reduce overall control efficiency.

This context calls for a strategic shift from reliance on single tactics toward integrated system management. We propose a “conservation-first, augmentation-supplemented” framework. This approach emphasizes enhancing on-farm habitat quality and biodiversity to bolster resident natural enemies while using commercially reared Trichogramma as a precision reinforcement tool during key vulnerable periods—such as early outbreak stages or when initiating control in new areas [44]. Scaling this approach through landscape-coordinated management—with unified monitoring and synchronized releases across 1–2 km zones—can significantly improve efficacy at ecologically meaningful scales [45].

The success of implementing such an integrated system depends on supportive policies, market recognition of ecosystem services, and a reliable supply of high-quality biological agents. As warmer springs advance pest phenology [46], maintaining intervention precision will increasingly require tools such as degree-day forecasting models and AI-assisted monitoring (e.g., image recognition for real-time population tracking) [47,48]. Life-cycle assessments indicate that such IPM systems can enhance agricultural sustainability and resilience while remaining economically viable [49]. Together, these advances pave the way for more adaptive, regionally tailored, and ecologically sound pest management strategies.

## Data Availability

The original contributions presented in this study are included in the article. Further inquiries can be directed to the corresponding authors.
